# Supplementary material for: Metabolic network analysis reveals microbial community interactions in anammox granules
Source: Nat Commun. 2017 May 31;8:15416. doi: 10.1038/ncomms15416 (PMC5460018; doi:10.1038/ncomms15416)
Supplement: Supplementary Information — Supplementary Figure and Supplementary Tables [file ncomms15416-s1.pdf]

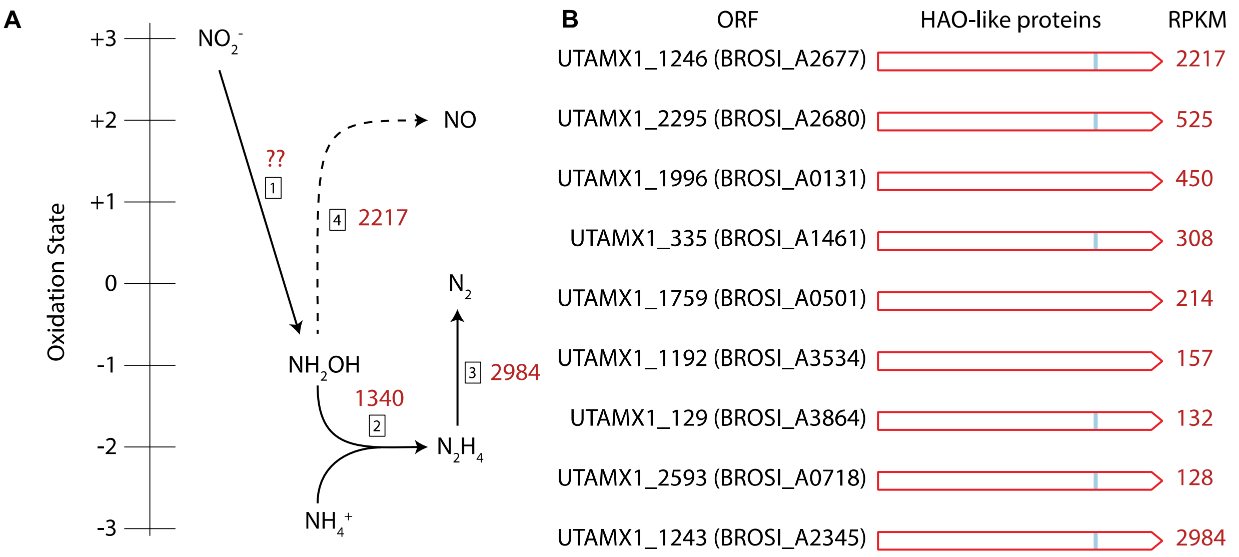


**Supplementary Figure 1.** A. Expression of genes involved in the anammox metabolic pathway recently proposed by Oshiki et al. (2016). Genes: [1] unidentified HAO-like protein involved in nitrite reduction, [2] hydrazine synthase subunit A, [3] hydrazine dehydrogenase, [4] hydroxylamine oxidoreducase (kust1061 ortholog). Red text indicates gene expression values (RPKM). B. HAO-like proteins identified in the UTAMX1 genome. '*Ca*. B. sinica' ortholog shown in brackets. Red text indicates gene expression values (RPKM). Blue line indicates cross-linking tyrosine residue.

**Supplementary Table 1.** Metagenomic and metatranscriptomic sequencing statistics.

| **Sample** | **DNA_09_04_13_S1** | **DNA_09_09_15** | **RNA_09_09_15** |
|---|---:|---:|---:|
| **total reads** | 4,123,584 | 45,715,259 | 22,056,078 |
| **filtered reads** | 4,116,701 | 36,946,335 | 18,437,366 |
| **merged reads** | 1,816,852 | 34,274,103 | 11,068,638 |
| **merged length** | 300-590 | 125-240 | 125-240 |
| **mRNA** | - | - | 8,780,658 |
| **rRNA** | - | - | 2,287,980 |
| **Description** | MiSeq metagenome | HiSeq metagenome | HiSeq metatranscriptome |
| **BioSample No.** | SAMN05785373 | SAMN05785375 | SAMN05785376 |

**Supplementary Table 2.** Metagenomic and metatranscriptomic read mapping statistics.

| MAG | genome size | read count | coverage (RPKM) | relative abundance | total reads (%) | mapped reads (%) | mRNA count | expression (RPKM) | relative expression | total mRNA (%) | mapped mRNA (%) |
|---|---|---|---|---|---|---|---|---|---|---|---|
| AMX1 | 3,142,066 | 19,308,825 | 185.41 | 62.0% | 56.3% | 58.6% | 3,057,308 | 153.72 | 54.8% | 34.8% | 47.2% |
| CHB1 | 2,424,598 | 4,872,772 | 62.29 | 20.8% | 14.2% | 14.8% | 1,099,805 | 71.16 | 25.4% | 12.5% | 17.0% |
| CHB4 | 1,189,945 | 677,700 | 17.25 | 5.8% | 2.0% | 2.1% | 49,708 | 6.44 | 2.3% | 0.6% | 0.8% |
| CHB5 | 1,684,758 | 490,899 | 9.02 | 3.0% | 1.4% | 1.5% | 37,025 | 3.48 | 1.2% | 0.4% | 0.6% |
| CHB2 | 4,062,427 | 782,510 | 5.40 | 1.8% | 2.3% | 2.4% | 131,573 | 5.41 | 1.9% | 1.5% | 2.0% |
| BCD1 | 3,757,364 | 509,896 | 4.14 | 1.4% | 1.5% | 1.5% | 130,039 | 5.33 | 1.9% | 1.5% | 2.0% |
| CFX1 | 2,777,515 | 274,783 | 3.14 | 1.1% | 0.8% | 0.8% | 148,199 | 9.00 | 3.2% | 1.7% | 2.3% |
| CFX2 | 2,924,761 | 260,104 | 2.71 | 0.9% | 0.8% | 0.8% | 47,987 | 2.49 | 0.9% | 0.5% | 0.7% |
| PRO2 | 2,355,954 | 252,380 | 1.99 | 0.7% | 0.7% | 0.8% | 129,168 | 5.21 | 1.9% | 1.5% | 2.0% |
| PRO1 | 3,818,019 | 123,619 | 1.59 | 0.5% | 0.4% | 0.4% | 67,184 | 4.32 | 1.5% | 0.8% | 1.0% |
| AMX2 | 3,437,337 | 190,916 | 1.68 | 0.6% | 0.6% | 0.6% | 84,967 | 3.98 | 1.4% | 1.0% | 1.3% |
| CFX3 | 2,655,948 | 100,255 | 1.14 | 0.4% | 0.3% | 0.3% | 31,808 | 1.73 | 0.6% | 0.4% | 0.5% |
| CFX5 | 3,776,973 | 150,431 | 1.21 | 0.4% | 0.4% | 0.5% | 23,103 | 0.98 | 0.3% | 0.3% | 0.4% |
| PLA1 | 4,435,946 | 152,921 | 1.04 | 0.3% | 0.4% | 0.5% | 105,111 | 3.72 | 1.3% | 1.2% | 1.6% |
| CPR1 | 892,243 | 8,398 | 0.27 | 0.1% | 0.0% | 0.0% | 9,895 | 1.64 | 0.6% | 0.1% | 0.2% |
| CFX4 | 4,654,268 | 35,973 | 0.23 | 0.1% | 0.1% | 0.1% | 36,599 | 1.19 | 0.4% | 0.4% | 0.6% |
| CHB3 | 2,943,821 | 11,249 | 0.11 | 0.0% | 0.0% | 0.0% | 3,767 | 0.19 | 0.1% | 0.0% | 0.1% |

Total DNA reads mapping to metagenomic assembly: 32,968,582 (96%)

Total mRNA reads mapping to metagenomic assembly: 6,473,379 (74%)

**Supplementary Table 3.** Metagenome-assembled genome (MAG) DDBJ/ENA/GenBank accession numbers.

| MAG | BioSample | GenBank Accession | Organism |
|---|---|---|---|
| AMX1 | SAMN06342761 | MWTF00000000 | Candidatus Brocadia sp. UTAMX1 |
| AMX2 | SAMN06342762 | MWTE00000000 | Candidatus Brocadia sp. UTAMX2 |
| BCD1 | SAMN06342764 | MWTC00000000 | Sphingobacteriales bacterium UTBCD1 |
| CFX1 | SAMN06342765 | MWTB00000000 | Anaerolineae bacterium UTCFX1 |
| CFX2 | SAMN06342766 | MWTA00000000 | Anaerolineae bacterium UTCFX2 |
| CFX3 | SAMN06342767 | MWSZ00000000 | Anaerolineae bacterium UTCFX3 |
| CFX4 | SAMN06342768 | MWSY00000000 | Chloroflexi bacterium UTCFX4 |
| CFX5 | SAMN06342769 | MWSX00000000 | Anaerolineae bacterium UTCFX5 |
| CHB1 | SAMN06342770 | MWSW00000000 | Ignavibacteriales bacterium UTCHB1 |
| CHB2 | SAMN06342771 | MWSV00000000 | Ignavibacteriales bacterium UTCHB2 |
| CHB3 | SAMN06342772 | MWSU00000000 | Ignavibacteriales bacterium UTCHB3 |
| CPR1 | SAMN06342775 | MWSR00000000 | Microgenomates bacterium UTCPR1 |
| PLA1 | SAMN06342763 | MWTD00000000 | Planctomycetes bacterium UTPLA1 |
| PRO1 | SAMN06342773 | MWST00000000 | Polyangiaceae bacterium UTPRO1 |
| PRO2 | SAMN06342774 | MWSS00000000 | Rhodocyclaceae bacterium UTPRO2 |
